# The Jefferson Scale of Empathy: a nationwide study of measurement properties, underlying components, latent variable structure, and national norms in medical students

**DOI:** 10.1007/s10459-018-9839-9

**Published:** 2018-07-02

**Authors:** Mohammadreza Hojat, Jennifer DeSantis, Stephen C. Shannon, Luke H. Mortensen, Mark R. Speicher, Lynn Bragan, Marianna LaNoue, Leonard H. Calabrese

**Affiliations:** 1 Center for Research in Medical Education and Health Care, Sidney Kimmel Medical College at Thomas Jefferson University, 1015 Walnut Street, Suite 320, Philadelphia, PA 19107 USA; 2 American Association of Colleges of Osteopathic Medicine, Bethesda, MD USA; 3 The Cleveland Clinic, Cleveland, OH USA

**Keywords:** Empathy, Factor analysis, Medical students, Psychometrics, National norms

## Abstract

The Jefferson Scale of Empathy (JSE) is a broadly used instrument developed to measure empathy in the context of health professions education and patient care. Evidence in support of psychometrics of the JSE has been reported in health professions students and practitioners with the exception of osteopathic medical students. This study was designed to examine measurement properties, underlying components, and latent variable structure of the JSE in a nationwide sample of first-year matriculants at U.S. colleges of osteopathic medicine, and to develop a national norm table for the assessment of JSE scores. A web-based survey was administered at the beginning of the 2017–2018 academic year which included the JSE, a scale to detect “good impression” responses, and demographic/background information. Usable surveys were received from 6009 students enrolled in 41 college campuses (median response rate = 92%). The JSE mean score and standard deviation for the sample were 116.54 and 10.85, respectively. Item-total score correlations were positive and statistically significant (*p *< 0.01), and Cronbach α = 0.82. Significant gender differences were observed on the JSE scores in favor of women. Also, significant differences were found on item scores between top and bottom third scorers on the JSE. Three factors of Perspective Taking, Compassionate Care, and Walking in Patient’s Shoes emerged in an exploratory factor analysis by using half of the sample. Results of confirmatory factor analysis with another half of the sample confirmed the 3-factor model. We also developed a national norm table which is the first to assess students’ JSE scores against national data.

## Introduction

### Personality in health professions education and patient care

The importance of professionalism and its assessment in physicians-in-training and in-practice (Stern 2006) has led to the acknowledgment that at least two major components are involved in medical education (Hojat et al. 2014). One component includes a set of “cognitive” abilities often reflected in academic attainment, performances on examinations of recalling factual information, declarative knowledge, and procedural skills. The other component, often described under the rubric of “personality” includes features such as personal qualities, attitudes, interests, values, and other psychosocial characteristics. In a paradigm of physician performance, both the cognitive abilities as well as personality are associated with patient outcomes, the ultimate goal of medical education (Gonnella et al. 1993).

### Empathy in health professions education and patient care

Empirical research suggests that a number of personality attributes, including empathy, are among significant predictors of clinical competence of physicians-in-training (Hojat et al. 2002a, 2014) and of patient outcomes (Hojat et al. 2011; Del Canale et al. 2012). Empathy has been described as one major element of professionalism in medicine (Veloski and Hojat 2006), and the most frequently mentioned personality attribute of the humanistic physician (Linn et al. 1987). Cultivating empathy is among the goals of medical education, endorsed by professional medical organizations. For example, the Medical Schools Objectives Project of the Association of American Medical Colleges (1999) includes enrichment of empathy among the educational objectives of medical schools. Also, in a position paper, the American Board of Internal Medicine (1983) recommended that humanistic qualities should be instilled and assessed as an essential part of physician education.

### Definition of empathy in health professions education and patient care

Despite consensus on the importance of empathy in patient care, there is no unanimity on the definition of empathy in the context of patient care (Matravers 2014). The historical ambiguity associated with the term and the lack of psychometrically sound instruments to measure empathy in that context contributed to a dearth of empirical research on empathy in medical education and patient care. Despite a lack of conceptual clarity, empathy has recently received considerable attention in public media, academia, national and international politics, business, arts, ethics, and particularly in health professions education and patient care (Coplan 2014).

Based on a comprehensive review of relevant literature, empathy in patient care was defined as a predominantly *cognitive* (rather than an affective or emotional) attribute that involves an *understanding* (rather than feeling) of pain and suffering of the patient, combined with a capacity to *communicate* this understanding, and an *intention to help* (Hojat 2016; Hojat et al. 2001). The four key terms in this definition are in italics to underscore their significance in the construct of empathy in patient care, and make a distinction between empathy and sympathy (which is defined as a predominantly emotional reaction). This distinction is important because empathy and sympathy have different consequences in clinical outcomes (see Hojat 2016, pp. 3–16, 71–81).

### Measurement of empathy in health professions education and patient care

Prior to the development of the JSE, no psychometrically sound instrument was available for measuring empathy in the context of health professions education and patient care. Several empathy measuring instruments, including the Interpersonal Reactivity Index (IRI, Davis 1983); the Empathy Scale (Hogan 1969); and the Emotional Empathy Scale (Mehrabian and Epstein 1972) were available and used by medical education researchers.

However, these instruments were developed for administration to the general population. None is specific enough to capture the essence of empathy in the context of health professions education and patient care (Evans et al. 1993). In other words, none of these instruments has “face” and “content” validity in that context (Hojat and Gonnella 2017; Hojat 2018). There was a need for a content-specific and context-relevant empathy measuring instrument. In response to that need, the *Jefferson Scale of Empathy* (*JSE*) was developed (Hojat et al. 2001, 2002b). The JSE is a 20-item instrument specifically developed to measure empathy in the context of health professions education and patient care for administration to health professions students and practitioners. Items are answered on a 7-point Likert-type scale (1 = Strongly Disagree, 7 = Strongly Agree). Half of the items are positively worded and directly scored, and the other half are negatively worded (reverse scored).

Three versions of the JSE are available. One version is used for administration to medical students (S-Version). The second version was developed for administration to practicing health professionals including physicians, nurses, dentists, pharmacologists, clinical psychologists, and other clinicians involved with patient care (HP-Version). The third version (HPS-Version) was developed for administration to all health professions students other than medical students. All three versions are very similar in content with only minor differences in a few words to adjust the instrument for its target population. The JSE has been recognized as the most researched and widely used instrument in medical education research (Colliver et al. 2010), and has been translated into 56 languages, and used in more than 80 countries (Hojat 2016). An abundance of evidence has been reported exclusively in samples of health professional students and practitioners in support of the psychometrics of the JSE by researchers in the United States and abroad (for a review see Hojat 2016, pp. 84–128, 276–286).

### Validity evidence in support of the JSE

Numerous empirical studies have been published in which associations between scores of the JSE and a number of pertinent variables have been reported. However, the litmus test for the validity of any empathy-measuring instrument in patient care is the evidence to show significant associations between scores of the instrument and indicators of positive patient outcomes for physicians-in-practice, and clinical competence in physicians-in-training. In addition, associations of the scores of the instrument with conceptually relevant personal qualities, and group differences in the expected direction can provide support for the validity of the instrument.

#### Patient outcomes

Significant associations between physicians’ scores on the JSE and tangible patient outcomes were confirmed in two empirical studies in the U.S. and Italy. In the first study in the U.S. (Hojat et al. 2011), electronic records of adult patients with diabetes mellitus were examined. Findings showed that patients of physicians who scored high on the JSE were significantly more likely to have good control of their disease (determined by hemoglobin A1c test result < 7.0%, and low-density lipoprotein cholesterol: LDL-C < 100) compared to patients of physicians who scored low on the JSE.

In a second large scale study in Palma, Italy (Del Canale et al. 2012) electronic records of adult patients with types 1 and 2 diabetes mellitus were examined and information on acute metabolic complications (e.g., diabetic ketoacidosis, coma, and hyperosmolar) that required hospitalization were extracted. Results showed statistically significant associations between physicians’ high scores on the JSE and lower rates of patients’ hospitalization.

#### Clinical competence

In a study of medical students (Hojat et al. 2002), a statistically significant association was found between the students’ JSE scores and the faculty’s global ratings of students’ clinical competence in core clerkships in family medicine, internal medicine, obstetrics and gynecology, pediatrics, psychiatry, and surgery. Also, significant associations were observed between students’ JSE scores and ratings of clinical competence given by standardized patients in 10 Objective Structured Clinical Examination (OSCE) stations (Berg et al. 2011, 2015).

In a study by LaNoue and Roter (2018) significant associations were found between self-reported JSE scores in third-year medical students, and observations of empathic communication pattern with simulated patients, measured by the Roter Interactional Analysis System (Roter and Larson 2002). In a large scale study at the Cleveland Clinic (Chaitoff et al. 2017), significant associations were observed between physicians’ JSE scores and standardized measures of patients’ communication experiences with their physicians.

#### Personality measures

In the first validity study of the JSE in medical students and internal medicine residents (Hojat et al. 2001), statistically significant correlations of moderate magnitude were found between JSE scores and the following variables: scores on the Empathic Concern and Perspective Taking scales of the Interpersonal Reactivity Index, personality facets of Warmth and Dutifulness on the NEO PI-R (Costa and McCrea 1992), and self-reported measures of Compassion and Sympathy (single items). In another study with internal medicine residents (Hojat et al. 2005a), significant correlations were observed between JSE scores and Perspective Taking, Empathic Concern, and Fantasy scales of the IRI, but not with the Personal Distress scale of the IRI (which is not conceptually relevant to empathy in patient care). Similar findings were reported by Costa and his colleagues (2017) with samples of medical students from five countries using different translated versions of the JSE.

In a study with medical students in the U.S. (Hojat et al. 2005b), higher scores on the JSE were associated with higher scores on Sociability. Scores of the JSE have also been linked to the “big five” personality factors such as Agreeableness, Openness to Experience, Conscientiousness, and Extraversion in medical students in Portugal (Costa et al. 2014).

Empirical evidence in studies with health professions students and practitioners showed that a number of personal quality measures that are conducive to relationship building were positively correlated with JSE scores, including emotional intelligence (Arora et al. 2010; Austin et al. 2005; Kliszcz et al. 2006); cooperativeness (Hong et al. 2011); desirable professional behavior (Brazeau et al. 2010); patient-centered care and orientation toward integrative patient care (Hojat et al. 2015a); positive social influence measured by peer nomination (Hojat et al. 2015b); and clinical and humanistic excellence measured by peer nomination (Pohl et al. 2011). Burnout resiliency measured by scores on the Personal Accomplishment of the MBI (Maslach 1993) was found to be inversely correlated with the JSE scores in medical students (Hojat et al. 2015c). The JSE scores were significantly associated with orientation toward teamwork and interprofessional collaboration in a study with allopathic medical students (Hojat et al. 2012), osteopathic medical students (Calabrese et al. 2013), nursing students (Ward et al. 2009), and pharmacy students (Van Winkel et al. 2011).

In other studies, scores of the JSE yielded negative correlations with personality attributes that are detrimental to positive interpersonal relationships, such as measures of aggression-hostility (Hasan et al. 2013; Hojat et al. 2005b) and indicators of burnout such as Depersonalization and Emotional Exhaustion (Hojat et al. 2015c; Lamothe et al. 2014; Zenasni et al. 2012). In a study with Chinese nursing students (Xia et al. 2011), an inverse relationship was found between the JSE scores and scores of the Neuroticism scale of the Eysenck Personality Questionnaire (Eysenck and Eysenck 1975).

### Validity by comparing contrasted groups

A measuring instrument is valid when it can demonstrate group differences in the expected direction. The expectations are based on previous research, theories, and behavioral tendencies described in the literature.

#### Gender difference

Some have suggested that women’s behavioral style is generally more “empathizing” than men (Baron-Cohen 2003). Indeed, in a majority of studies, female health professions students and practicing clinicians obtained significantly higher JSE mean scores than their male counterparts (Hojat et al. 2001, 2002a, b; Alcorta-Garza et al. 2005; Fjortoft et al. 2011). In a large scale study of 11 entering classes of medical students, women’s JSE mean scores were higher than men in all classes, and the gender difference was statistically significant for 10 out of the 11 classes (Hojat and Gonnella 2015). Several plausible explanations have been given for gender differences in empathy, including social learning, genetic predisposition, evolutionary underpinnings, and other factors (for a review see Hojat 2016, pp. 169–187).

#### Specialty interest

In her doctoral dissertation, Bailey (2001) reported that medical students who planned to pursue a career in specialties requiring extensive and prolonged encounters with patients received significantly higher scores on empathy measured by the IRI (Davis 1983) than their counterparts who planned to pursue procedure-oriented specialties. Given the aforementioned finding, it can be hypothesized that high scorers on the JSE are more inclined to choose specialties that require continuous and prolonged encounters with patients. These specialties are described as “people-oriented” such as primary care specialties (general internal medicine, family medicine, and pediatrics) and psychiatry. Conversely, it can be predicted that low scorers on the JSE would be more interested in specialties that often require less interaction with patients and often involve diagnostic or therapeutic procedures. These specialties are described as “technology/procedure-oriented” such as hospital-based specialties (pathology, radiology, and anesthesiology), urology, surgery and surgical subspecialties.

The aforementioned hypotheses were confirmed in a number of studies. For example, physicians in the “people-oriented” specialties scored higher on the JSE compared to others in “technology-/procedure-oriented” specialties (Hojat et al. 2002b), consistent with findings reported by others in the United States and abroad (Chen et al. 2007, 2012; Kataoka et al. 2012; Voinescu et al. 2009). In a study with first-year medical students, the JSE was administered at the beginning of medical school before students were exposed to formal medical education (Hojat et al. 2005b). Findings showed a significant association between JSE scores and specialty interest in favor of those planning to pursue “people-oriented” specialties. Interestingly, the pattern of malpractice claims against physicians in different specialties has proven to be consistent with research findings on the JSE scores so that those practicing in “people-oriented” specialties were less likely charged with malpractice litigation (Taragin et al. 1994). However, such associations between specialty interest and JSE scores were not observed in osteopathic medical students (Calabrese et al. 2013) .

### Study purposes

In a majority of the studies in which the JSE has been used, research participants were students from allopathic medical schools; only a few studies with osteopathic medical students are available. To fill the gap, this study was designed to examine some psychometric aspects of the JSE in a nationwide sample of osteopathic medical students and investigate associations between JSE scores and a number of selected demographic and background variables. Another goal was to develop the first national norm table for the assessment of the JSE scores in the corresponding population. The present study is unique for two reasons. First, it is the first nationwide study in which the JSE was used, and second, it is the first psychometric study of the JSE in osteopathic medical students. Medical education in osteopathic medical schools (that grant the DO degree), as compared to that in allopathic medical schools (that grant the MD degree) places more emphasis on a holistic approach to patient care, and the integration of the entire body systems. The osteopathic manipulative treatment (OMT) is a unique feature of the osteopathic medical education that involves using the hands for diagnosis and treatment of illnesses and injuries. This is part of a larger nationwide Project in Osteopathic Medical Education and Empathy (POMEE) with a broader scope to provide national norms for the JSE scores and examine associations between empathy in medical students and demographic variables, undergraduate major, specialty interest, and prior health care employment. Also, the study will explore changes in empathy as students progress through medical school (Newton 2018).  

## Methods

### Participants

Participants included 6009 first-year students (53% men, *n *= 3175) from 41 (out of 44) campuses of colleges of osteopathic medicine in the U.S., representing 93% of all branch campuses of colleges of osteopathic medicine.

### Measures

The web-based survey included the JSE-S Version, the “Infrequency” Scale of the Zuckerman-Kuhlman Personality Questionnaire (ZKPQ, Zuckerman 2002), and demographic and background information. The landing page of the survey depicted a photo of an elderly patient and a female clinician holding hands in an empathic manner, followed by a page with a brief description of the project and the importance of the study, and another page with a brief message signed by the dean of the student’s corresponding college to encourage their participation.

Because most items of the JSE are transparent, respondents can answer the survey in a way that they consider to be more socially acceptable. One approach to control for the confounding effect of making “good impression” responses is to measure the degree of such attempts. For that purpose, we used the “Infrequency” scale of the ZKPQ. This is a 10-item scale (True/False responses) that was developed to measure the degree of “good impression” response bias. Scores on this scale higher than 3 indicate questionable validity of the respondent’s record. This scale has previously been used with medical students to control for the tendency to make “good impression” responses (Hojat 2016; Hojat et al. 2005b).

### Procedures

We developed a preliminary version of the survey instrument that went through several iterations in pilot testing. Participants of the pilot studies included some members of research teams and their colleagues at Jefferson (the headquarters of the project), and the AACOM, and volunteer students from two medical schools.

Arrangements were made to select one (and sometimes two) research coordinators from each participating college, at senior administrator or faculty level, to serve as a liaison between the college, AACOM, and Jefferson research teams. The contribution of these research coordinators proved to be of utmost importance in encouraging students to participate and increasing response rates. The study was approved by the Institutional Review Board of Thomas Jefferson University and each participating college.

### Statistical analyses

Correlational analysis, exploratory and confirmatory factor analysis, *t* test, Chi-square test, and analysis of variance and covariance were used in statistical analyses of data. When appropriate, effect size estimates were calculated to examine the practical significance of statistically significant findings.

## Results and discussion

### Response rates and usable sample

Response rates for the 41 participating college campuses ranged from a low of 33% to a high of 100%, with a median of 92% and a mean of 85%. Response rates for 80% of participating colleges exceeded our 75% target goal. This pattern of high response rates in a national study using an online survey is very impressive and probably unique. Response rates in online survey administration have been reported to hover around 35% in a meta-analytic study (Cho et al. 2013). A total of 6146 students responded. However, 137 were excluded due to incomplete data; thus, complete usable data were available for 6009 students.

### Demographic information

Of the 6009 students in the usable sample, 3175 (53%) were men, 2795 (47%) were women; and the rest (*n *= 39, < 1%) either declined to answer or indicated other choices. Gender composition in this sample is very close to that for the total population of the 2017 first-year matriculants (*n*=7197) in the U.S. colleges of osteopathic medicine (54.7% men, 45.3% women) (AACOM 2018). Students’ age ranged from 19 to 51 years, with a mean of 24.8, median of 24, and a standard deviation of 3.4 years. The mean age for the total 2017 matriculants in U.S. colleges of osteopathic medicine was 24 years, and age range was 19-51 (AACOM 2018). The majority of respondents were White/Caucasian (*n *= 3618, 60%), followed by Asian (*n *= 1627, 27%), Hispanic/Latino/Spanish (*n *= 337, 6%), and Black/African American (*n *= 216, 4%). Ethnic composition of our study sample was similar to that of the total population of the 2017 first-year matriculants in U.S. colleges of osteopathic medicine (59.8% White/Caucasian, 24.4% Asian, 7.5% Hispanic/Latino, 3.2% Black/African American) (AACOM 2018).

### Descriptive statistics

Descriptive statistics, and Cronbach’s coefficient α for the JSE for men and women separately, and for both genders combined are reported in Table 1. The JSE mean score for the combined sample was 116.54 (*SD *= 10.85). The mean score for men was 114.40 (*SD *= 11.34, range = 26–140) and for women it was 118.78 (*SD *= 9.78, range = 69–140). The gender difference was statistically significant (*F*_(1,5968)_ = 250.94, *p *< 0.0001). Results remained unchanged by using analysis of covariance, controlling for the Infrequency scores as a covariate (adjusted mean for men = 114.44, for women = 118.74; adjusted *F*_(1,5967)_ = 241.72, *p *< 0.0001).

**Table 1:** Descriptive statistics of the JSE scores in a nationwide sample of first-year students at the beginning of academic year from 41 campuses of colleges of osteopathic medicine in the United States

Statistics	Men (*n* = 3175)	Women (*n* = 2795)	Total (*n *= 5970)^a^
Mean	114.40	118.78	116.54
Median	115	120	117
Mode	116	119	119
SD	11.34	9.78	10.85
Possible range	20–140	20–140	20–140
Actual range	26–140	69–140	26–140
Skewness	− 0.54	− 0.61	− 0.60
Kurtosis	1.29	0.58	1.15
Cronbach’s coefficient alpha	0.83	0.81	0.82

^a^Thirty-nine respondents who did not specify their gender or reported other gender categories were excluded from analysis in this table

In national and international studies, the reported JSE mean scores vary, mostly hovering around 112 (standard deviations around 12) (Hojat 2016, pp. 124, 275–331). In one large scale study of first-year students at one allopathic medical school in the U.S. (*n *= 2637) who completed the JSE at the beginning of medical school, a mean score of 114.3 (*SD *= 10.4) was reported (Hojat and Gonnella 2015). Although the difference between the JSE mean scores obtained in the present study and in the study of allopathic medical students is statistically significant in the favor of osteopathic medical students (*t*_(8605)_ = 8.54, *p* < 0.001), the effect size estimate of the difference (*d *= 0.20) indicates that such a difference is not practically (clinically) important (Cohen 1987).

The skewness index for the total study sample was − 0.60. Kurtosis for the JSE score distribution was 1.15. Similar findings have been reported in a large sample of allopathic medical students in one U.S. medical school (Hojat and Gonnella 2015). Cronbach’s coefficient α was 0.82 (0.83 for men and 0.81 for women). In examining a large number of national and international studies in which the JSE was used, the alpha coefficients were mostly in the 0.70–0.80 range with an average of 0.78 (Hojat 2016, pp. 124, 275–331). The alpha coefficients ranged from 0.75 to 0.84 (average = 0.80) for 11 entering classes between 2002 and 2012 in a study with allopathic medical students (Hojat and Gonnella 2015). These findings suggest similarity in reliability coefficients of the JSE in both osteopathic and allopathic medical students.

### Item statistics

Respondents used the full range of responses (1–7) for all items. Item mean scores ranged from a low of 3.52 to a high of 6.63 (median = 5.92). Item standard deviations ranged from 0.63 to 1.50 (median = 1.07). The pattern of findings is similar to that reported for a large sample of first-year students in an allopathic medical school (Hojat and Gonnella 2015).

### Item-total score correlations

Pearson correlation coefficients were calculated to examine correlations between each item score and the total score of the JSE. For that purpose, we calculated the corrected item-total score correlations (by excluding the corresponding item from the total JSE score). Corrected item-total score correlations ranged from a low of 0.20 to a high of 0.61 (median = 0.46). All correlations were positive and statistically significant (*p *< 0.01). Summary results are reported in Table 2. The pattern of findings is similar to that reported for first-year students in an allopathic medical school (Hojat and Gonnella 2015).

**Table 2:** Corrected item-total score correlations^a^ and effect size estimates of item discrimination indices^b^ for the Jefferson Scale of Empathy in a national sample of 6009 first-year students at the beginning of academic year from 41 campuses of colleges of osteopathic medicine in the United States

Abbreviated JSE Items^c^	Corrected item-total score correlation	Effect size of discrimination index
Understanding emotions in patient-clinician relationship (16)	0.61	1.42
Empathy as a therapeutic factor (20)	0.60	1.37
Attention to patients’ personal experiences (8)	0.54	1.30
Non-verbal cues and body language in understanding patients (13)	0.53	1.29
Patient-physician emotional ties in medical treatment (11)	0.53	1.24
Place of emotion in medical treatment (14)	0.53	1.14
Understanding is therapeutic to patient (10)	0.51	1.27
Standing in patients’ shoes (9)	0.51	1.27
Life events in understanding physical complaints (12)	0.49	1.33
Attention to patients’ emotions (7)	0.47	1.13
Empathy and clinical success (15)	0.45	1.18
Understanding makes patients feel better (2)	0.43	1.02
Thinking like patients for better care (17)	0.41	1.14
Understanding body language in communication (4)	0.38	0.98
Understanding patients’ feelings influences treatment (1)	0.38	1.03
Taking patients’ perspectives (6)	0.26	0.81
Viewing patients’ perspectives (3)	0.26	0.78
Enjoy literature and arts (19)	0.25	0.77
Sense of humor and clinical outcomes (5)	0.21	0.67
Physician influenced by patients’ personal bonds (18)	0.20	0.67
Mean (median)	0.43 (0.46)	1.09 (1.14)

^a^Correlations between scores on each item and the JSE total score by excluding the corresponding item from the total score. All correlations are statistically significant (*p* < 0.01)

^b^In calculation of the effect size estimate (Cohen’s *d*) of the discrimination index, the item mean score of the JSE high scorers (top 33%, *n *= 2096), was subtracted from the item mean score of the JSE low scorers (bottom 33%, *n *= 2028), divided by the pooled standard deviation of the corresponding item

^c^Numbers in parentheses correspond to the item numbers in the JSE

### Item discrimination effect size indices

To address the discrimination power of each item, we calculated the item discrimination index. For that purpose, we divided the sample into two groups of approximately top-third high scorers on the JSE (score > 122, *n *= 2096) and bottom-third low scorers (JSE score < 112, *n *= 2028). For each item, we calculated the difference of item mean scores between the top and bottom-third scoring groups, then divided the mean difference for each item by the pooled standard deviation of the item scores to calculate the item discrimination effect size index (similar to the Cohen’s *d,* Cohen 1987) (item discrimination effect size index = *M*_top-third_ − *M*_bottom-third_/pooled *SD*).

Effect size estimates of the item discrimination indices are reported in Table 2. The item discrimination effect size indices ranged from a low of 0.67 to a high of 1.42 (median = 1.14). According to operational definitions on the magnitude of effect size estimates (*d*), suggested by Cohen (1987), all of the item discrimination effect size estimates reported in Table 2 are considered substantial and practically important. As expected, the item-total score correlations and the effect size estimates of the item discrimination indices were highly correlated (*r *= 0.97, *p* < 0.0001). The pattern of findings is similar to that reported in a large sample of first-year students in an allopathic medical school (Hojat and Gonnella 2015).

### Factor analyses

We conducted both exploratory and confirmatory factor analyses of the JSE. For that purpose, we randomly divided the sample into two groups. Data for the first group (*n *= 3004) were used for exploratory factor analysis to examine underlying components of the JSE. Data for the second group (*n *= 3005) were used for confirmatory factor analysis to confirm the latent variable structure of the JSE. As expected, no significant difference was observed between the two above-mentioned groups on age, gender, ethnicity/race, or their scores on the JSE and the Infrequency scale of the ZKPQ.

### Exploratory factor analysis

In most of the factor analytic studies of the JSE, orthogonal (varimax) rotation has been used to obtain independent factors. In this study we used oblique rotation (promax) to allow correlations among the extracted factors in order to examine if factor patterns remain unchanged. We also limited the number of retained factors to three to make the findings comparable to the previous studies in allopathic medical students and physicians (Hojat 2016; Hojat et al. 2002; Hojat and LaNoue 2014; Costa et al. 2017). The scree test was used to determine the number of factors to retain before rotation and showed that the plot of eigenvalues levels off after extraction of the third factor. This supported our decision to retain three factors for rotation. The Kaiser–Meyer–Olkin measure for sampling adequacy (MSA) was used which resulted in an overall index of 0.90, supporting the adequacy of data for factor analysis. Also, the Bartlett’s test for sphericity showed that the intercorrelation matrix was factorable (χ^2^_(133)_ =  924.11, *p* < 0.0001).

The eigenvalues for the three retained factors before rotation were 5.52, 1.66, and 1.39, accounting for 28, 8, and 7% of the total variance, respectively. The first factor, entitled “Perspective Taking” in previous studies, included 10 items with factor coefficients equal to or greater than 0.35 (Table 3). The Cronbach’s coefficient alpha for this factor was 0.80. The second factor, “Compassionate Care,” included six items with factor coefficients equal to or greater than 0.52. The Cronbach’s coefficient alpha for this factor was 0.71. The third factor, “Walking in Patient’s Shoes,” included only two items with factor coefficients of 0.77 and 0.72. The Cronbach’s coefficient alpha for this factor was 0.71. This last factor may be considered as a residual factor because according to Velicer and Fava (1998), a minimum number of three items per factor is required for a stable factor.

**Table 3:** Rotated factor pattern for the Jefferson Scale of Empathy using a national sample of first-year students at the beginning of academic year from 41 campuses of colleges of osteopathic medicine in the United States (*n *= 3004)

Abbreviated JSE Items^b^	Factors^a^		
	Factor 1	Factor 2	Factor 3
Standing in patients’ shoes (9)	**0.66**	− 0.03	0.00
Understanding is therapeutic to patient (10)	**0.62**	0.01	0.01
Understanding emotions in patient-clinician relationship (16)	**0.61**	0.16	0.00
Thinking like patients for better care (17)	**0.60**	− 0.08	− 0.03
Non-verbal cues and body language in understanding patients (13)	**0.57**	0.10	0.00
Empathy as a therapeutic factor (20)	**0.54**	0.20	0.00
Understanding makes patients feel better (2)	**0.53**	0.01	− 0.01
Understanding body language in communication (4)	**0.48**	− 0.04	0.07
Empathy and clinical success (15)	**0.44**	0.10	0.00
Sense of humor and clinical outcomes (5)	**0.35**	− 0.09	− 0.01
Attention to patients’ personal experiences (8)	0.03	**0.65**	− 0.02
Patient-physician emotional ties in medical treatment (11)	0.03	**0.64**	− 0.01
Place of emotion in medical treatment (14)	0.03	**0.62**	0.00
Understanding patients’ feelings influences treatment (1)	− 0.09	**0.59**	− 0.08
Life events in understanding physical complaints (12)	0.05	**0.54**	0.06
Attention to patients’ emotions (7)	0.03	**0.52**	0.07
Enjoy literature and arts (19)	− 0.02	0.28	0.05
Physician influenced by patients’ personal bonds (18)	− 0.01	0.21	0.05
Taking patients’ perspectives (6)	− 0.03	0.05	**0.77**
Viewing patients’ perspectives (3)	0.03	− 0.01	**0.72**
Eigenvalues	**5.52**	**1.66**	**1.39**

Principal component factor analysis with oblique rotation used for half of the sample (*n *= 3004). Confirmatory factor analysis was performed in the other half of the sample

^a^Items are listed by the descending order of magnitude of factor coefficients with each factor. Factor coefficients > 0.35 are shown in bold. Items were scored using a 7-point Likert-type scale. Half of the items are reverse scored

^b^Numbers in parentheses refer to the item number in the JSE

Two items had moderate factor coefficients (0.21 and 0.28) on the second factor (see Table 3). Both of these items showed significant item-total score correlations and substantial discrimination effect size indices, indicating that these items significantly predict the JSE total score and can make a distinction between high and low JSE scorers. Therefore, despite their lower factor coefficients, we suggest to retain them in the instrument.

The three aforementioned factors emerged in a multinational-multilanguage study by Costa and his colleagues (2017) who examined the underlying constructs of the Interpersonal Reactivity Index (IRI) and the Jefferson Scale of Empathy (JSE) in samples of medical students in five countries. The general pattern of findings in this exploratory factor analysis is similar to those in most other studies in the U.S. and abroad. For example, similarities in factor pattern are observed in studies reported for allopathic medical students (Hojat and LaNoue 2014), physicians (Hojat et al. 2002) and nurses (Ward et al. 2009) in the Unites States and for samples of physicians in Italy (DiLillo et al. 2009), medical students in Iran (Shariat and Habibi 2013), Korea (Roh et al. 2010), Japan (Kataoka et al. 2009), Mexico (Alcorta-Garza et al. 2005), South Africa (Vallabh 2011), mainland China (Wen et al. 2013), Taiwan (Hsiao et al. 2012), Brazil (Paro et al. 2012), Austria (Preusche and Wagner-Menghin 2013), and England (Tavakol et al. 2011). In particular, the two factors of “Perspective Taking” and “Compassionate Care” emerged in almost all of the factor analytic studies of the JSE.

### Confirmatory factor analysis

**Fig. 1:**
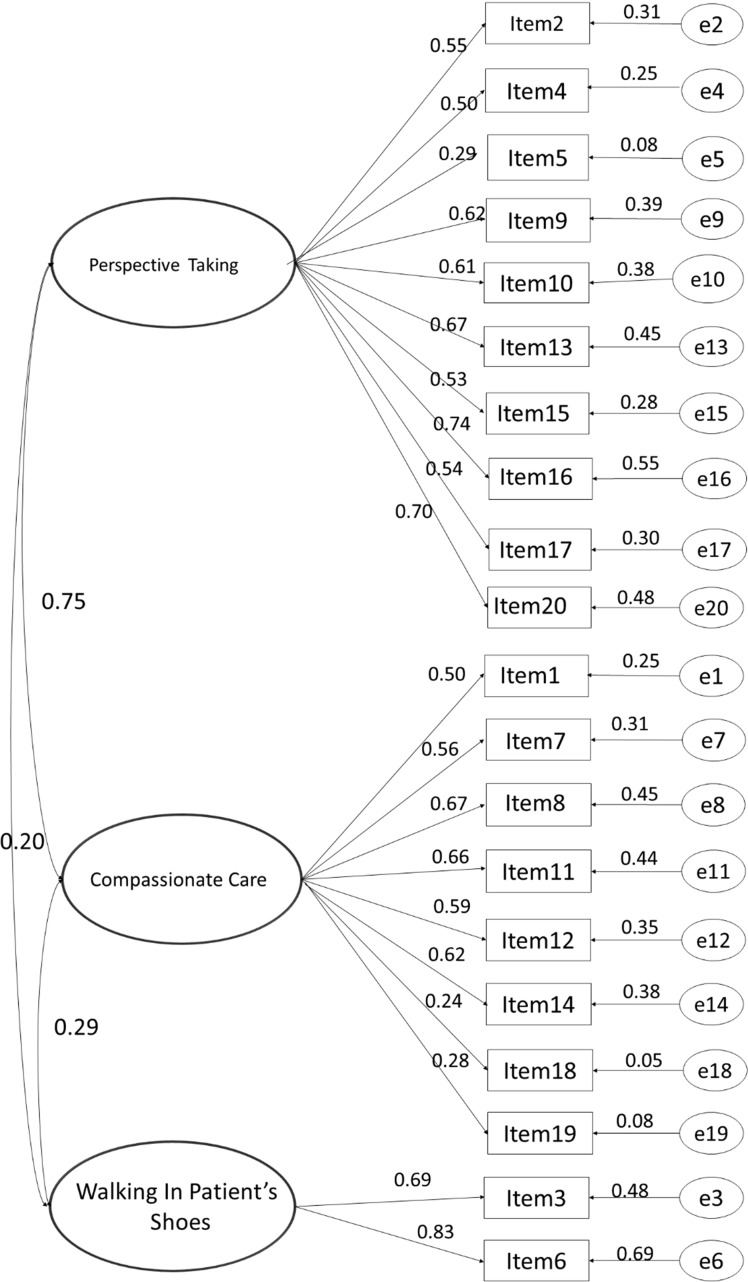
Three-factor model (latent variable structure) of the Jefferson Scale of Empathy

We used confirmatory factor analysis in a structural equation modeling (SEM) framework to confirm the JSE’s latent variable structure (Arbuckle and Wothke 1999). Based on the findings from exploratory factor analysis, we specified the measurement model as follows: The 20 items comprising the JSE were modeled as resulting from one of three underlying latent variables: “Perspective Taking” (10 items), “Compassionate Care” (8 items), and “Walking in Patient’s Shoes” (2 items) (see Fig. 1). The regression coefficient for one item-to-latent variable path for each latent variable was set to 1.0 to scale the latent variable, and covariances among the latent variables were modeled. A total of 43 parameters were estimated. The model was identified with 167 degrees of freedom.

An ‘independence’ model was also estimated, where all indicators load to a single latent variable as a first step in establishing evidence for dimensional structure, for comparison to the measurement model (Model 1, null model in Table 4). Data were imputed using a person-level mean imputation for three cases,  *n *= 3005. The data showed marked multivariate non-normality, Mardia’s multivariate kurtosis = 208.39 (critical ratio value = 192.45).

**Table 4:** Summary Results of Confirmatory Factor Analysis Fit Statistics (*n *= 3005)

(Fit Reference Value)	Model 1 Null Model	Model 2 3 Factor Model	Model 3 2 Factor Model
*Χ*^2^ (*p *> 0.05)	15,695.36, *p* < .001	1343.56, *p* = .002 (Bollen–Stine)	1229.473, *p* < .001
*χ*^2^/*df* (~ <5.0)	82.61	8.05	9.17
RMSEA ~ (< 0.05)	0.165	0.048	0.052
CFI*	0.0	0.925	0.922
TLI*	0.0	0.914	0.911

*Values > 0.90 are considered good, and values > 0.95 are excellent

#### Estimation

We assume that the JSE is a reflexive indicator of an underlying continuous latent variable and thus conduct this analysis in a ‘normal theory’ framework. Simulation research suggests that in cases of continuous but highly non-normal data, parameter estimates using maximum likelihood (ML) estimation may be approximately correct in large samples, but that the standard errors estimated are likely too small (Curran et al. 1996). To account for the non-normality of the multivariate distribution, we report estimation results and their significances with robust standard errors and a corrected model test statistic; the Bollen–Stine bootstrapped *p* value for the Chi-square test for model fit. Finally, given the large sample size, we also estimated the model using an asymptotically distribution free approach (ADF) which does not require an assumption of multivariate normality. This approach is used only to provide supplementary information for a more complete picture of model fit. Parameter estimates and significances (Fig. 1) are those from the bootstrapped ML estimation.

#### Model fit

In SEM, it is known that the Chi-square fit statistic is often positively biased using the maximum likelihood (ML) estimator when the data are non-normal (Curran et al. 1996). We therefore report the Bollen–Stine bootstrapped *p* value for Chi-square obtained from AMOS as a measure of exact fit. However, it is also well known that the Chi-square statistics are sensitive to sample size and that large samples can produce significant Chi-square values (indicating misfit) even when fit is acceptable. The Bollen–Stine bootstapped *p* value is not immune to this issue. Thus, we also report other well accepted measures of model fit. The model Chi-square divided its degrees of freedom is a parsimony adjusted transformation of the Chi-square value to account for model complexity. The RMSEA (root-mean squared error) is also a measure of exact fit and indexes how well the model fits the population covariance matrix. A perfect fitting model would have RMSEA of 0, and therefore smaller values are desired. The comparative fit index (CFI) is an incremental measure of model fit relative to the null model and can be loosely interpreted as a measure of proportion of variance explained. Finally, we report the Tucker-Lewis Index (TLI), an additional incremental fit index related to the CFI, but that is parsimony adjusted.

The 3-factor measurement model (Model 2, Table 4) fit reasonably well with RMSEA = 0.048 and the ratio of Chi-square to *df* = 8.0. Both the TLI and the CFI exceeded 0.90 (0.925 and 0.914, respectively) indicating good, but not an excellent fit (Table 4). The model estimated with the asymptotically distribution free estimation method showed increased fit with respect to the RMSEA, but worse fit with the CFI and TLI.

Modification indices suggest that correlations among errors as well as correlations between error terms and latent variables are likely responsible for model misfit rather than misspecification of the measurement model overall. The only modification indices suggested that bear on the model structure were for indicator latent relationships in the WIPS factor. As previously investigated (Hojat and LaNoue 2014), a two-factor model (owing to the fact that the WIPS factor only has two indicators) was again investigated by specifying a model where the WIPS latent variable and the two items that load on it are omitted (Model 3, 2-factor model in Table 4). This model is a worse fit to the data (Table 4).

In a large scale exploratory and confirmatory factor analytic study with allopathic medical students (Stansfield et al. 2016), a 3-factor model was confirmed as an acceptable fit for the preclinical years, but a 4-factor model emerged as a better fit for the clinical years of medical school education. Similarly, the 3-factor structure was also confirmed in medical students from two Spanish medical schools (Ferreira-Valente et al. 2016), and in Turkish medical students (Bilgel and Ozcakir 2017).

In summary, data in this large scale study with osteopathic medical students supported most of the previously reported findings on underlying constructs and confirmation of the latent variable structure of the JSE (S-Version). Similarities in factor pattern of the JSE in different samples in different medical education systems, and in different countries indicate that the underlying components of the scale are relatively stable, regardless of cultural variation. The three components of “Perspective Taking”, “Compassionate Care”, and “Walking in the Patient’s Shoes” which emerged in this and some other factor analytic studies of the JSE are consistent with the components of empathy often reported in the literature.

### National norm table

Prior to this study, there were no national norm tables available for the JSE or for any other empathy measuring instrument in medical students. This project provided a golden opportunity to develop the first and only national norm table of empathy scores to determine the percentile rank of any first-year student enrolled in osteopathic medical colleges in the U.S. at the beginning of the academic year, prior to being exposed to formal medical education

There was a total of 5818 first-year students (3071 men and 2747 women) with scores on the JSE after excluding those students who did not identify as “male” or “female” and whose scores were above the cutoff on the Infrequency Scale of the ZKPQ; thus, students who attempted to produce socially desirable responses were excluded from the national norm table. We calculated percentile ranks for raw scores of the JSE from the score distribution in the national sample. Results are presented in Table 5. Because gender differences in the JSE scores (in favor of women) have consistently been observed in a large number of studies (for a review see Hojat 2016, pp. 169–187) including this study, we separately calculated the percentile ranks for men and women.

**Table 5:** National norm table for the Jefferson Scale of Empathy (JSE-S Version) (for first-year students at the beginning of academic year from 41 campuses of colleges of osteopathic medicine in the United States)

JSE	Men (*n *= 3071)			Women (*n *= 2747)			Men and women combined (*n *= 5818)		
Raw score	*f*	*cf*	Percentile rank	*f*	*cf*	Percentile rank	*f*	*cf*	Percentile rank
≤ 80	19	19	< 1	5	5	< 1	24	24	< 1
81–82	12	31	1	2	7	< 1	14	38	1
83–84	7	38	1	1	8	< 1	8	46	1
85–86	8	46	1	3	11	< 1	11	57	1
87–88	8	54	2	5	16	< 1	13	70	1
89–90	24	78	2	5	21	1	29	99	1
91–92	26	104	3	5	26	1	31	130	2
93–94	34	138	4	8	34	1	42	172	3
95–96	31	169	5	21	55	2	52	224	3
97–98	70	239	7	30	85	3	100	324	5
99–100	79	318	9	35	120	4	114	438	7
101–102	102	420	12	34	154	5	136	574	9
103–104	125	545	16	70	224	7	195	769	12
105–106	139	684	20	78	302	10	217	986	15
107–108	183	867	25	110	412	13	293	1279	19
109–110	199	1066	31	115	527	17	314	1593	25
111–112	211	1277	38	153	680	22	364	1957	31
113–114	218	1495	45	147	827	27	365	2322	37
115–116	221	1716	52	204	1031	34	425	2747	44
117–118	203	1919	59	207	1238	41	410	3157	51
119–120	208	2127	66	237	1475	49	445	3602	58
121–122	198	2325	72	211	1686	58	409	4011	65
123–124	175	2500	79	212	1898	65	387	4398	72
125–126	144	2644	84	203	2101	73	347	4745	79
127–128	119	2763	88	206	2307	80	325	5070	84
129–130	89	2852	91	161	2468	87	250	5320	89
131–132	92	2944	94	117	2585	92	209	5529	93
133–134	59	3003	97	82	2667	96	141	5670	96
135–136	37	3040	98	48	2715	98	85	5755	98
137–138	16	3056	99	28	2743	99	44	5799	99
139–140	15	3071	> 99	4	2747	> 99	19	5818	> 99

Excluded were respondents who did not select “male” or “female” (< 1%), and those who did not answer all items of the Infrequency Scale of the ZKPQ (used to identify respondents who attempted to make “good impression”). Only 2.5% of respondents scored above the cutoff score of > 3 on the Infrequency Scale who were excluded from data used for this norm table

f: Frequency

cf: Cumulative frequency

National norm data reported in Table 5 can be used only for the assessment of JSE scores of new matriculants to osteopathic medical schools. We are in the process of collecting similar data from nationwide samples of students in different years of osteopathic medical schools that will be reported in due course.

## Conclusion

Findings of this study with first-year students at U.S. colleges of osteopathic medicine provided additional evidence in support of credibility of the JSE. Findings reaffirmed the latent variable structure of the JSE. Results were generally similar to those reported for allopathic medical students and other health professions students and practitioners. The norm table developed in this study can assist in assessing individuals’ scores against national norms, and can potentially serve as an additional criterion for admissions decisions, or for breaking ties in applicants with similar academic qualifications.
